# 

**Published:** 1854-07

**Authors:** 


Vol. VII. JULY, 1854. NO. 4.

### THE DENTAL REGISTER OF THE WEST.

EDITED BY

JAMES TAYLOR, M. D., D. D. S.

PUBLISHED QUARTERLY

AT $2 PER ANNUM.

AGENTS.

J. B. Dunlevy, Pittsburgh, Pa.

Dr. T. S. Rives, St. Louis, Mo.

Messrs. Jones, White & McCurdy, Phila. New York city, and Boston, Mass.

Dr. J. M. Brown, Cincinnati.

Solomon Brown, New York city

John Klein, Phil., Pa.

CINCINNATI:
PRINTED BY T. WRIGHTSON, 167 WALNUT STREET.
1854.

